# Bayesian Inference for Duplication–Mutation with Complementarity Network Models

**DOI:** 10.1089/cmb.2015.0072

**Published:** 2015-11-01

**Authors:** Ajay Jasra, Adam Persing, Alexandros Beskos, Kari Heine, Maria De Iorio

**Affiliations:** ^1^ Department of Statistics & Applied Probability, National University of Singapore, Singapore, Singapore.; ^2^ Department of Statistical Science, University College London, London, United Kingdom.

**Keywords:** duplication–mutation with complementarity (DMC) model, particle marginal Metropolis–Hastings (PMMH), protein–protein interaction (PPI) network, sequential Monte Carlo (SMC)

## Abstract

**We observe an undirected graph *G* without multiple edges and self-loops, which is to represent a protein–protein interaction (PPI) network. We assume that *G* evolved under the duplication–mutation with complementarity (DMC) model from a seed graph, *G*_0_, and we also observe the binary forest Γ that represents the duplication history of *G*. A posterior density for the DMC model parameters is established, and we outline a sampling strategy by which one can perform Bayesian inference; that sampling strategy employs a particle marginal Metropolis–Hastings (PMMH) algorithm. We test our methodology on numerical examples to demonstrate a high accuracy and precision in the inference of the DMC model's mutation and homodimerization parameters.**

## 1. Introduction

As a result of breakthroughs in biotechnology and high-throughput experiments thousands of regulatory and protein–protein interactions have been revealed, and genome-wide protein–protein interaction (PPI) data are now available. Protein–protein interactions are one of the most important components of biological networks, as they are fundamental to the functioning of cells. To gain a better understanding of why these interactions take place, it is necessary to view them from an evolutionary perspective. The evolutionary history of PPI networks can help answer many questions about how present-day networks have evolved and provide valuable insight into molecular mechanisms of network growth (Kreimer et al., 2008; Pereira-Leal et al., 2007). However, inferring network evolution history is a statistical and computational challenging problem as PPI networks of extant organisms provide only snapshots in time of the network evolution. There has been recent work on reconstructing ancestral interactions (e.g., Dutkowski and Tiuryn, 2007; Gibson and Goldberg, 2009; Patro et al., 2012). The main growth mechanism of PPI network is gene duplication and divergence (mutations) (Wagner, 2001); all proteins in a family evolve from a common ancestor through gene duplications and mutations, and the protein network reflects the entire history of the genome evolution (Vazquez et al., 2003). In this article we follow Li et al. (2013), and we develop computational methods to infer the growth history and the parameters under the given model incorporating not only the topology of observed networks, but also the duplication history of the proteins contained in the networks. In their article, Li et al. (2013) propose a maximum likelihood approach. The authors establish a neat representation of the likelihood function, and it is this representation that is used in this article. The duplication history of the proteins can be inferred independently by phylogenetic analysis (Patro et al., 2012; Pinney et al., 2007).

The approach we adopt here is first to obtain a numerically stable estimate of the likelihood function, under fixed parameters; this is achieved via the sequential Monte Carlo (SMC) method (see Doucet et al., 2000, and Gordon et al., 1993). This approach can then be used to infer the parameters of the model, from a Bayesian perspective, as well as the growth history, via a Markov chain Monte Carlo (MCMC) method. To the best of our knowledge, this has not been considered in the literature, although related ideas have appeared for simpler models in Wang et al. (2014). Our computational strategy not only improves on likelihood estimation in comparison to Li et al. (2013), but also provides a natural setup to perform posterior inference on the parameters of interest.

This article is structured as follows. In section 2, we detail the model and associated computational method for statistical inference. In section 3, our numerical results are presented. In section 4, the article is concluded with some discussion of future work.

## 2. Model and Methods

We follow similar notation and exposition as in Li et al. (2013) to introduce the protein–protein interaction network, its duplication history, and the duplication–mutation with complementarity (DMC) model (Vazquez et al., 2003). In particular, the notions of adjacency and duplication are made concrete there. We also introduce the associated Bayesian inference problem with which this work is primarily concerned (i.e., that of inferring the parameters of the DMC model). We then describe a particle marginal Metropolis–Hastings (PMMH) algorithm (Andrieu et al., 2010) that can be used to perform such inference.

### 2.1. PPI network and DMC model

Consider an undirected graph *G* without multiple edges and self-loops, where the nodes represent proteins and the edges represent interactions between those proteins. Such a graph is called a PPI network, and as in Li et al. (2013), we denote the vertex set by *V*(*G*), the edge set by *E*(*G*), and the number of nodes in *G* by |*V* (*G*)|. All nodes that are adjacent to a node *v* (not including *v* itself) comprise the neighborhood of *v*, and that neighborhood is denoted by *N_G_*(*v*).

We assume that *G* evolved from a seed graph *G*_0_ via a series of duplication, mutation, and homodimerization steps under a DMC model. Under the DMC model, at each time step *t*, the graph *G_t_* evolves from *G_t_*_−1_ by the following processes in order:

1. The anchor node *u_t_* is chosen uniformly at random from *V* (*G_t_*_−1_), and a duplicate node *v_t_* is added to *G_t_*_−1_ and connected to every member of $N_{G_{t - 1}} ( u_t )$. This is the duplication step, and it yields an intermediary graph denoted $G_{t - 1}^*$.
2. For each $w \in N_{G_{t - 1}^*} ( u_t )$, we uniformly choose one of the two edges in $\{  ( u_t , w ) , ( v_t , w ) \}  \subseteq E ( G_{t - 1}^* )$ at random and delete it with probability (1 − *p*). This is the mutation step, and the parameter *p* is henceforth referred to as the mutation parameter.
3. The anchor node *u_t_* and the duplicate node *v_t_* are connected with probability *p_c_* to finally obtain *G_t_*. This is the homodimerization step, and the parameter *p_c_* is henceforth known as the homodimerization parameter.

The DMC model is Markovian, and we denote the transition density at time *t* (which encompasses the three aforementioned steps) by $p_{ \cal M} ( G_t\  \mid\   G_{t - 1} )$, where ${ \cal M}  :  = ( p , p_c )$. If we assign to a seed graph some prior density $p_{ \cal M} ( G_0 )$, then the density of the observed graph *G* will be

\begin{align*}p_{ \cal M} ( G ) \,= \sum_{{ \cal H} \backslash \{
G_n \} } \left[ p_{ \cal M} ( G_0 ) \prod_{t = 1}^n p_{ \cal M} (
G_t\ \mid \ G_{t - 1} ) \right] , \tag{1}\end{align*}

where $G = G_n , n = \mid V ( G ) \mid - \mid V ( G_0 ) \mid$, and ${ \cal H} = ( G_0 , G_1 , \ldots , G_n = G )$ denotes the collection of growth histories. In this work, a seed graph will always be the graph consisting of two connected nodes; thus, $\mid V ( G_0 ) \mid = 2$ and $p_{ \cal M} ( G_0 ) = 1$. Note that we are summing over all possible growth histories by which *G* can evolve from a seed graph. Also note that a growth history ${ \cal H}$ induces a unique sequence of duplicate nodes, $\theta ( { \cal H} ) = ( v_1 , \ldots , v_n )$ (Li et al., 2013).

### 2.2. Bayesian inference

In practice, one will not have access to the parameters (*p*, *p_c_*), and they must be inferred given *G*. Thus, in the Bayesian setting, our objective is to consider the posterior density

\begin{align*}\pi ( { \cal M} \ \mid \ G ) \propto p ( { \cal M} ) p_{ \cal M} ( G ) , \tag{2}\end{align*}

where $p ( { \cal M} )$ is some proper prior for (*p*, *p_c_*) that we assume can easily be computed (at least pointwise up to a normalizing constant).

The total number of growth histories grows exponentially with *n* (Li et al., 2013), and so any computations involving (1), and thus (2) [e.g., the evaluation of $p_{ \cal M} ( G )$], could potentially become very expensive. In the following sections, we reformulate the inference problem in the same manner as in Li et al. (2013) to alleviate this issue.

### 2.3. Duplication history

As in Li et al. (2013), let Γ be a binary forest, that is, a collection of rooted binary trees. The authors of Li et al. (2013) describe a scheme that encodes the duplication history of a growth history ${ \cal H}$ within a series of duplication forests, $( \Gamma_0 , \Gamma_1 , \ldots , \Gamma_n )$, where each forest Γ_*t*_ corresponds to a graph *G_t_*. We describe that scheme here.

Consider a trivial forest Γ_0_, whose only two isolated trees each consist of a single node. Each of those isolated nodes will correspond to a node within the seed graph *G*_0_. To build Γ_1_ from Γ_0_, one replaces an anchor node *u*_1_ from Γ_0_ with a subtree, $\{  u_1 , v_1 \}$, consisting of two leaves (*v*_1_ is the duplicate node including *G*_1_ but not *G*_0_). This process continues until one builds the series of forests $( \Gamma_0 , \Gamma_1 , \ldots , \Gamma_n = \Gamma )$ to correspond to ${ \cal H}$.

**FIG. 1.**
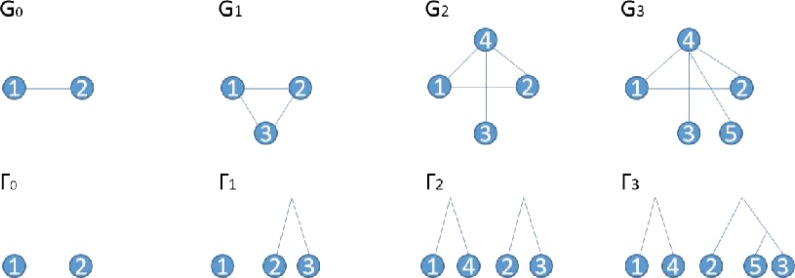
An example growth history for a network together with the corresponding history of the duplication forest. In this example, (*u*_1_, *u*_2_, *u*_3_) = (2, 1, 3) and (*v*_1_, *v*_2_, *v*_3_) = (3, 4, 5).

As highlighted in Li et al. (2013), the duplication forest Γ (corresponding to *G*) is uniquely determined by ${ \cal H}$ and a list of anchor nodes, $\pi = ( u_1 , \ldots , v_n )$. The important thing to emphasize here is that given the duplication forest Γ and *G*, one now only needs to infer the duplication nodes sequence $\theta ( { \cal H} ) = ( v_1 , v_2 , \ldots , v_n )$ to reconstruct the complete growth history ${ \cal H}$. For instance, at the first step backward, knowledge of Γ_*n*_ = Γ together with *v_n_* uniquely identifies the anchor node *u_n_*, thus one can reconstruct *G_n_*_−1_ and Γ_*n*−1_; this is then repeated for the remaining backward steps. Thus, given $\theta ( { \cal H} )$, one can construct the growth history ${ {\cal H}}$ backward-in-time using the backward operators defined in section 2.4 of Li et al. (2013), which constructs *G_t_*_−1_, Γ_*t*−1_ deterministically given (*G_t_*, Γ_*t*_, *v_t_*), for $t = n , n - 1 , \ldots , 1$. An example of a growth history is given in Figure 1.

### 2.4. Bayesian inference given the duplication history

Now suppose that in addition to *G*, a practitioner is given Γ corresponding to *G*. Our new objective—and the primary inference problem with which this work is concerned—is to consider the posterior density $\pi ( { \cal M} \mid G , \Gamma )$. Notice that we have the joint distribution:

\begin{align*}\pi ( { \cal M} , \{  G , \Gamma \}  , \theta ) = p ( { \cal M} ) p_{ \cal M} ( G_0^{ \theta} , \Gamma_0^{ \theta} ) \prod_{t = 1}^n p_{ \cal M} ( G_t^{ \theta} , \Gamma_t^{ \theta} \ \mid \ G_{t - 1}^{ \theta} , \Gamma_{t - 1}^{ \theta} )\end{align*}

where $\theta = ( v_1 , \ldots , v_n )$ is a sequence of duplication nodes compatible with the observed *G,* Γ, and $G_0^{ \theta} , \Gamma_0^{ \theta} , \ldots , G_n^{ \theta} , \Gamma_n^{ \theta}$ the corresponding reconstructed history. We are thus interested in the parameter posterior:

\begin{align*}
\begin{split} \pi ( { \cal M} \ \mid \ G , \Gamma ) & \propto p ( { \cal M} ) p_{ \cal M} ( G , \Gamma ) , \\ p_{ \cal M} ( G , \Gamma ) & = \sum_{{ \theta} \mid G , \Gamma} \left[ p_{ {\cal M}} ( G_0^{ \theta} , \Gamma_{0}^{ \theta} ) \prod_{t = 1}^{n} p_{ {\cal M}} ( G_t^{ \theta} , \Gamma_{t}^{ \theta} \mid G_{t - 1}^{ \theta} , \Gamma_{t - 1}^{ \theta} ) \right] , \end{split}
 \tag{3}\end{align*}

The density $p_{ {\cal M}} ( G_0 , \Gamma_0 )$ is typically a trivial term that can be ignored in practice. As the duplication forest Γ limits the number of allowable anchor-and-duplicate node pairs, one can see that the number of possible growth histories is reduced.

### 2.5. Methods

We will now present an SMC algorithm that can sample the latent growth histories from the DMC model given the fixed parameters ${ {\cal M}}  :  = ( p , \ p_c )$. We then show that this algorithm can be employed within a PMMH algorithm, as in Andrieu et al. (2010), to sample from the posterior (3) and infer ${ {\cal M}}$ (and even *θ*|*G, *Γ).

An SMC algorithm simulates a collection of *N* samples (or, particles) sequentially along the index *t* via importance sampling and resampling techniques to approximate a sequence of probability distributions of increasing state-space, which are known pointwise up to their normalizing constants. In this work, we use the SMC methodology to sample from the posterior distribution of the latent duplication history:

\begin{align*}p_{ {\cal M}} ( \theta \ \mid \ G , \Gamma ) \propto p_{ {\cal M}} ( G_0^{ \theta} , \Gamma_{0}^{ \theta} ) \prod_{t = 1}^{n}p_{ {\cal M}} ( G_t^{ \theta} , \Gamma_{t}^{ \theta} \ \mid \ G_{t - 1}^{ \theta} , \Gamma_{t - 1}^{ \theta} )\end{align*}

backward along the index *t* via Algorithm 1 in the Appendix. The technique provides an unbiased estimate of the normalizing constant (Theorem 7.4.2 of Del Moral, 2004), $p_{ {\cal M}} ( G , \Gamma )$:

\begin{align*}\hat { p } _ { { \cal M } } ( G , \Gamma ) = \prod_ { t = 0 } ^ { n - 1 } \bigg [ \frac { 1 }  { N } \mathop \sum \limits_ { i = 1 } ^N W_t^i \bigg ] , \tag { 4 } \end{align*}

where each $W_t^i$ is an unnormalized importance weight computed in Algorithm 1. Note that under assumptions on the model, if *N > cn* for some *c <*∞ , then the relative variance of the estimate is ${ {\cal O}}$(*n/N*) (see Cérou et al., 2011). It is remarked that, as in Wang et al. (2014), one could also use the discrete particle filter (Fearnhead, 1998), with a possible improvement over the SMC method detailed in Algorithm 1 (see Wang et al., 2014, for some details).

This SMC can be employed within a PMMH algorithm to target the posterior of ${ {\cal M}}$ in (3). One can think of the deduced method as an MCMC algorithm running on the marginal ${ {\cal M}}$-space, but with the SMC unbiased estimate $\hat{p}_{ {\cal M}} ( G , \Gamma )$ replacing the unknown likelihood $\hat{p}_{ {\cal M}} ( G , \Gamma )$. More analytically, we can consider all random variables involved in the method and write down the equilibrium distribution in the enlarged state space, with ${ {\cal M}}$-marginal the target posterior $p_{ {\cal M}} ( G , \Gamma )$. Following Andrieu et al. (2010) and letting $\phi^i_t$ denote a sample (*G_t_*, Γ_*t*_) at time *t*, the extended equilibrium distribution is written as:

\begin{align*}
\begin{split} \pi^N & \left( l , { { \cal M } } , a_ { 1:n - 1 }
^ { 1:N } , \phi_ { 0:n - 1 } ^ { 1:N } \ \mid \ G , \Gamma
\right) = { \frac { \pi \left( { { \cal M } } , \phi_ { 0:n - 1 }
^ { l } \mid G , \Gamma \right) }  { N^ { n } } } \cdot { \frac {
\Psi_ { { \cal M } } ( a_ { 1:n - 1 } ^ { 1:N } , \phi_ { 0:n - 1
} ^ { 1:N } ) }  { q_ { { \cal M } } ( v_n^ { a_ { n - 1 } ^l } )
\left( \prod_ { t = 1 } ^ { n - 1 } { w } _ { t } ^ { a_ { t } ^l
} q_ { { \cal M } } ( v_t^ { a_ { t - 1 } ^l } ) \right) } } ,
\end{split}\tag{5}
\end{align*}

where $\Psi_{ {\cal M}}$ is the probability of all the variables associated to Algorithm 1, with $a_k^j , l \in \{  1 , .., N \}$ and the *φ*'s being the simulated variables at each step of Algorithm 1.

A PMMH algorithm (see Algorithm 2) samples from (5), and one can remove the auxiliary variables from the samples to obtain draws for the parameters from (3). Furthermore, one could even save the sampled growth histories with particle index *l* to obtain draws from the joint posterior $\pi ( { {\cal M}} , \theta \ \mid \ G , \Gamma )$. However, in this work, we are primarily interested in the inference of ${ {\cal M}}$.

## 3. Results

The variance of the estimate (4) plays a crucial role in the performance of Algorithm 2, as (4) is used to compute the acceptance probability within the PMMH algorithm. Thus, we first tested the variability of (4) as computed by Algorithm 1 to understand how the variance changes with |*V* (*G*)|. We then ran Algorithm 2 to sample from the posterior (3) and infer ${ {\cal M}}$ for a given pair of observations (*G*, Γ). We present the details of those experiments below.

### 3.1. Variance of $\hat{p}_{ {\cal M}} ( G , \Gamma )$

**APPENDIX FIG. A1.**
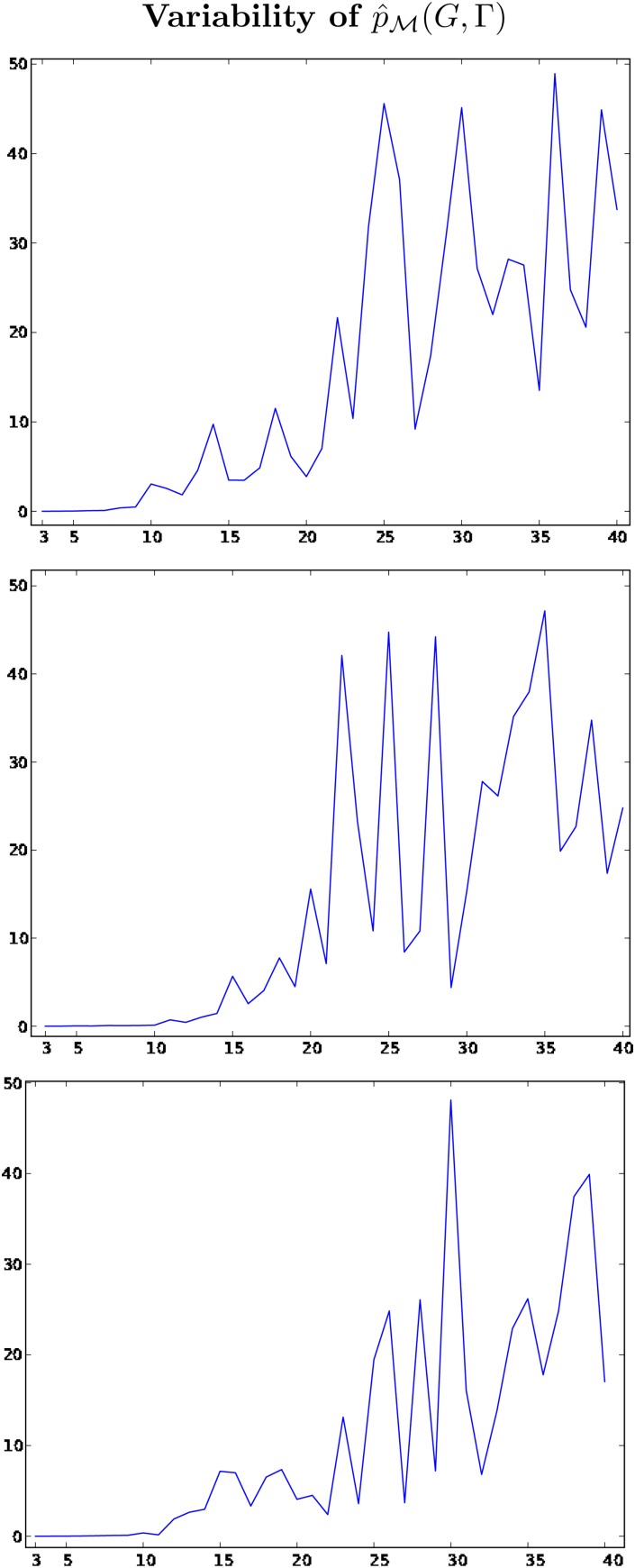
All plots illustrate the relative variance of $\hat{p}_{ {\cal M}} ( G_t , \Gamma_t )$, per |*V* (*G_t_*)| on the horizontal axis; the relative variance is the variance divided by the square of the expected value. In the top plot, the number of SMC particles used to compute each $\hat{p}_{ {\cal M}} ( G_t , \Gamma_t )$ is |*V*(*G_t_*)| * 5. In the middle and bottom plots, that number is |*V*(*G_t_*)| * 10 and |*V*(*G_t_*)| * 20, respectively. Recall that the seed graph, *G*_0_, has two nodes, and note that we did not compute $\hat{p}_{ {\cal M}} ( G_0 , \Gamma_0 )$ because it is trivial.

We simulated a graph *G* and a forest Γ from the DMC model with the parameters set as (*p* = 0.7, *p_c_* = 0.7), where |*V*(*G*)| = 40. We saved each pair (*G_t_*, Γ_*t*_) for 1 ≤ *t* ≤ 40, and we ran Algorithm 1 50 times per pair (with *N* = |*V*(*G_t_*)| * 5) to compute 50 unbiased estimates of $p_{ {\cal M}} ( G_t , \Gamma_t )$for 1 ≤ *t* ≤ 40. In the top of Appendix Figure A1 in Appendix A, we plot the relative variance of the estimate (or, the variance divided by the square of the expected value) per each value of |*V*(*G_t_*)|. We repeated the experiment two more times, with *N* = |*V*(*G_t_*)| * 10 and *N* = |*V*(*G_t_*)| * 20, and the associated output is also presented in Appendix Figure A1.

As remarked above, if *N > cn* for some *c < * ∞ , then the relative variance of $\hat {p}_{ {\cal M}} ( G_t , \Gamma_t )$ is ${ {\cal O}} ( n / N )$. Appendix Figure A1 confirms that the variance increases linearly, and that increasing the value of *N* with |*V*(*G*)| (at least linearly) will help to control the variance. However, the plots show that the relative variance is still high, which means that *N* will have to be large to ensure satisfactory performance of the PMMH in practice.

### 3.2. Parameter inference

We separately simulated a graph *G* and a forest Γ from the DMC model with the parameters again set as (*p* = 0.7, *p_c_* = 0.7), and we set |*V*(*G*)| = 15. Given only (*G*, Γ), we inferred (*p*, *p_c_*) with each parameter having a uniform prior on the interval [0.1, 0.9]. We set the number of particles within Algorithm 1 to be *N* = 2,000, and we ran the PMMH algorithm to obtain 10,000 samples from the extended target.

**APPENDIX FIG. A2.**
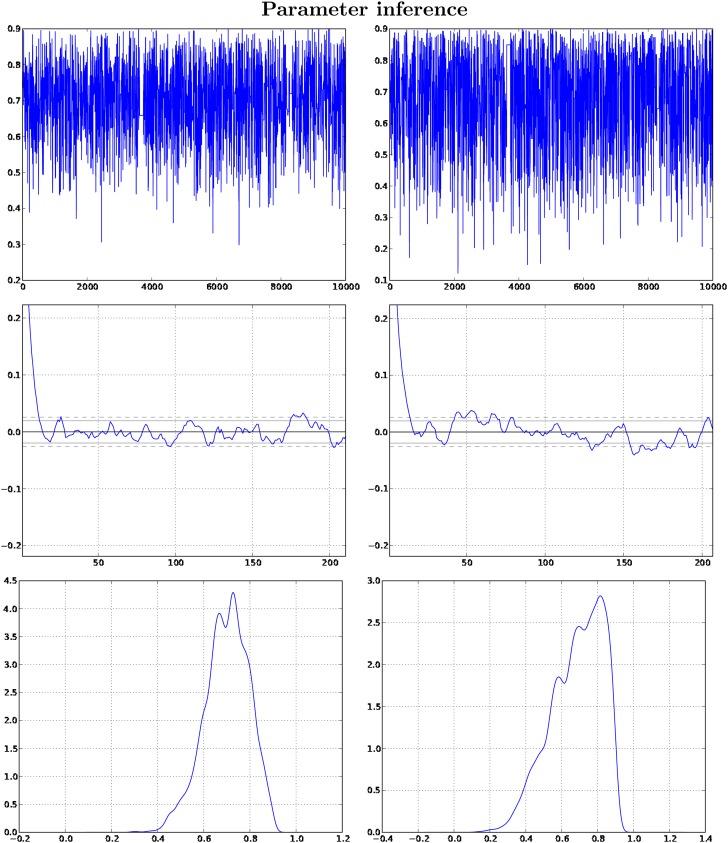
Plots associated with *p* and *p_c_* are at left and right respectively. The top figures are trace plots, with PMMH iteration running along the horizontal axes and parameter value running along the verticals. The middle figures are plots of the autocorrelation functions (with lag running along the horizontal axes), and at the bottom we present the parameter posterior densities.

Appendix Figure A2 in Appendix A illustrates good mixing of the PMMH algorithm and accurate inference of the parameters (*p*, *p_c_*). The trace plots show that the algorithm is not sticky, and the autocorrelation functions give evidence to an approximate independence between samples. The posterior densities are also interesting, in that they are clearly different from the uniform priors, and they show that the PMMH algorithm spends a majority of the computational time sampling the true parameter values.

## 4. Discussion

We have introduced a Bayesian inferential framework for the DMC model, where, as in Li et al. (2013), one assumes the pair (*G*, Γ) is observed and the parameters (*p*, *p_c_*) are unknown. We then described how an SMC algorithm can be used to simulate growth histories and ultimately be employed within PMMH to target the posterior distribution of the parameters (3), thereby opening up the possibility of performing Bayesian inference on the DMC model.

Numerical tests demonstrated that Algorithm 1 can have a high variability when |*V*(*G*)| is large and *N* is not sufficiently high, and this limits the scope of the inference problem, which can be tackled using the complete Algorithm 2. However, the proposals used in the example experiments within Algorithms 1 and 2 are naive, as the method simply chooses a candidate duplicate node $v_t^i$ at random from all permitted nodes given the current $G_t^i$, $\Gamma_t^i$. It is reasonable to assume that more sophisticated proposal densities could reduce the variance of the SMC and/or improve the mixing of the PMMH, thereby allowing one to perform inference when |*V*(*G_t_*)| is large and *N* is smaller. This could be explored in a future work.

## 5. Appendix A

## 6. Appendix B: Algorithm Summaries

**Algorithm 1:** Sequential Monte Carlo (SMC)

• Step 0: Input an observed graph *G* = *G_n_* and a corresponding observed forest Γ = Γ_*n*_, where *G* is not a seed graph.
• Step 1: Set *t* = *n*. For $i \in \{ 1 , \ldots , N \}$, sample a subtree with two nodes uniformly at random from $\Gamma_t^{i}$, and choose one of the two nodes uniformly as the proposed duplicate node $v_t^{i}$ (thus the other will be the anchor node). Using the backward operators defined in [10, section 2.4], construct each $( G_{t - 1}^i , \Gamma_{t - 1}^i )$ from the subtrees and $( G_t^i , \Gamma_t^i )$. For $i \in \{ 1 , \ldots , N \}$, compute the unnormalized weight
$$ { W } _ { t - 1 } ^i = { \frac { p_ { { \cal M } } ( G_t^ { i } , \Gamma_ { t } ^ { i } \mid G_ { t - 1 } ^i , \Gamma_ { t - 1 } ^i ) } { q_ { { \cal M } } ( v_t^i ) } } ,$$
where $q{ {\cal M}}$ is the density of the proposal mechanism used to sample $\{ u_t^i \}$.
• Step 2: If $\{ G_{t - 1}^{1:N} \}$ are not seed graphs, then set *t* = *t* − 1 and continue to Step 3. Otherwise, the algorithm terminates.
• Step 3: For $i \in \{ 1 , \ldots , N \}$, sample $a_{t}^i \in \{ 1 , \ldots , N \}$ from a discrete distribution on {1*, … ,N*} with *j*^th^ probability $w_t^j$ ∝ $W_t^j$. The sample $\{ a_{t}^{1:N} \}$ are the indices of the resampled particles. Set all normalized weights equal to *N*^−1^.
• Step 4: For $i \in \{ 1 , \ldots , N \}$, sample a subtree with two nodes uniformly at random from the resampled forest $\Gamma_t^{a_t^i}$, and select uniformly one of the two nodes as the proposed duplicate node $v_t^i$. Construct $( G_{t - 1}^i , \Gamma_{t - 1}^i )$ from $v_t^{i} , ( G_{t}^{a_t^i} , \Gamma_{t}^{a_t^i} )$. For $i \in \{ 1 , \ldots , N \}$, compute the unnormalized weight
$$ { W } _ { t - 1 } ^i = { \frac { p_ { { \cal M } } ( G_ { t } ^ { a_t^i } , \Gamma_ { t } ^ { a_t^i } \ \mid \ G_ { t - 1 } ^i , \Gamma_ { t - 1 } ^i ) } { q_ { { \cal M } } ( v_t^i ) } } .$$
Return to Step 2.

**Algorithm 2:** Particle Marginal Metropolis—Hastings (PMMH)

• Step 0: Set *r* = 0. Sample ${ {\cal M}}^{ ( r ) } \sim p ( \cdot )$. All remaining random variables can be sampled from their full conditionals defined by the target (5):
- Sample $\phi_{0:n - 1}^{ ( r ) , 1:N} , a_{1:n - 1}^{ ( r ) , 1:N} \sim \Psi_{{ {\cal M}}^{ ( r ) }} ( \cdot )$ via Algorithm 1.
- Choose a particle index $l^{ ( r ) } \propto W_0^{ ( r ) , l^{ ( r ) }}$.
Finally, calculate $\hat {p}_{{ {\cal M}}^{ ( r ) }} ( G , \Gamma )$ via (4).
• Step 1: Set *r* = *r* + 1. Sample ${ {\cal M}}^{*} \sim q ( \cdot \mid { {\cal M}} )$. All remaining random variables can be sampled from their full conditionals defined by the target (5):
- Sample $\phi_{0:n - 1}^{* , 1:N} , a_{1:n - 1}^{* , 1:N} \sim \Psi_{{ {\cal M}}^{*}} ( \cdot )$ via Algorithm 1.
- Choose a particle index $l^{*} \propto W_0^{* , l^{*}}$.
Finally, calculate $\hat{p}_{{ {\cal M}}^{*}} ( G , \Gamma )$ via (4).
• Step 2: With acceptance probability
$$1 \wedge { \frac { \hat { p } _ { { { \cal M } } ^ { * } } ( G , \Gamma ) q ( { { \cal M } } \mid { { \cal M } } ^ { * } ) } { \hat { p } _ { { { \cal M } } ^ { ( r - 1 ) } } ( G , \Gamma ) q ( { { \cal M } } ^* \mid { { \cal M } } ) } } ,$$
set $\Big ( l^{ ( r ) } , { {\cal M}}^{ ( r ) } , \phi_{0:n - 1}^{ ( r ) , 1:N} , a_{1:n - 1}^{ ( r ) , 1:N} \Big ) = \Big ( l^* , { {\cal M}}^* , \phi_{0:n - 1}^{* , 1:N} , a_{1:n - 1}^{* , 1:N} \Big )$. Otherwise, set
$$\Big ( l^{ ( r ) } , { {\cal M}}^{ ( r ) } , \phi_{0:n - 1}^{ ( r ) , 1:N} , a_{1:n - 1}^{ ( r ) , 1:N} \Big ) = \Big ( l^{ ( r - 1 ) } , { {\cal M}}^{ ( r - 1 ) } , \phi_{0:n - 1}^{{ ( r - 1 ) } , 1:N} , a_{1:n - 1}^{{ ( r - 1 ) } , 1:N} \Big )$$
Return to the beginning of Step 1.
